# Indicator condition-guided HIV testing in patients with mononucleosis-like illness and community-acquired pneumonia in an Emergency Department setting: A prospective study from Poland (2015–2018)

**DOI:** 10.1371/journal.pone.0354485

**Published:** 2026-07-29

**Authors:** Karolina Pyziak-Kowalska, Dorthe Raben, Ann Sullivan, Maksymilian Bielecki, Andrzej Horban, Justyna D. Kowalska

**Affiliations:** 1 Hospital for Infectious Diseases, Warsaw, Poland; 2 CHIP, Rigshospitalet, University of Copenhagen, Copenhagen, Denmark; 3 Directorate of Sexual Health and HIV Medicine, Chelsea and Westminster NHS Foundation Trust, London, United Kingdom; 4 Institute of Psychology, SWPS University, Warsaw, Poland; 5 Infectious Diseases Observational Research Unit, Medical University of Warsaw, Warsaw, Poland; 6 HIV Out-Patient Clinic, Hospital for Infectious Diseases, Warsaw, Poland; Pescara General Hospital, ITALY

## Abstract

**Background:**

Despite advances in HIV care, late diagnosis remains a major public health challenge in Europe, with nearly half of HIV diagnoses in the EU/EEA occurring at a late stage. In Poland, a substantial proportion of people living with HIV remain undiagnosed. Indicator condition–guided testing has been proposed as an effective strategy to reduce missed opportunities for earlier diagnosis.

**Methods:**

A prospective cohort study was conducted at the Emergency Department of the Hospital for Infectious Diseases in Warsaw, Poland, between May 2015 and March 2018, as part of the European OptTEST project. HIV DUO testing was offered to patients aged 18–65 years presenting with mononucleosis-like illness (MONO) or community-acquired pneumonia (CAP). We evaluated the prevalence of HIV infection and assessed linkage to HIV care among those testing positive. Confirmatory testing, CD4 cell counts at diagnosis, and linkage-to-care outcomes were collected.

**Results:**

Among 885 patients with indicator conditions (MONO: 794; CAP: 91), 636 (71.9%) accepted HIV testing. Twenty-two patients (3.5%) were confirmed HIV-positive. HIV prevalence was 2.6% among patients with MONO and 11.1% among those with CAP. Most HIV-positive patients were male (90.9%), with 59% identifying as men who have sex with men or bisexual. The median age was 32.5 years. The median CD4 cell count at diagnosis was 341 cells/µL overall and was lower in patients presenting with CAP than MONO (61 vs. 366.5 cells/µL). All patients diagnosed with HIV were linked to care, and 95.5% initiated antiretroviral therapy, with a median time to treatment of 8 days.

**Conclusions:**

HIV seroprevalence among patients presenting with MONO or CAP was higher than the national average, particularly among those with CAP. These findings support routine indicator condition–guided HIV testing in Emergency Departments to improve early HIV diagnosis and timely linkage to care.

## Introduction

Late diagnosis of HIV infection remains a critical public health challenge across Europe. According to the latest surveillance data from the European Centre for Disease Prevention and Control (ECDC) and WHO Regional Office for Europe, 48% of all HIV diagnoses in the EU/EEA in 2024 occurred at a late stage (CD4 count <350 cells/µL), while across the broader WHO European Region this proportion reached 54% [1]. Late diagnosis is associated with increased HIV-related morbidity and mortality, poorer response to antiretroviral therapy (ART), increased healthcare costs, and continued HIV transmission [2,3].

In Poland, the epidemiological situation presents particular challenges. In 2022, 2,604 new HIV infections were diagnosed (6.88 per 100,000 population), representing a 78.2% increase compared to the previous year, which coincided with increased population mobility following the conflict in Ukraine [4]. However, it is estimated that approximately 50% of people living with HIV in Poland remain undiagnosed [5]. Recent studies from Polish centres confirm persistently high rates of late presentation, with 53–63% of newly diagnosed patients presenting with CD4 counts below 350 cells/µL [6,7].

To address the challenge of undiagnosed HIV infection, the HIV in Europe initiative proposed indicator condition (IC)-guided testing as an innovative approach to expand HIV testing in healthcare settings [8]. The European HIDES I/II studies (HIV Indicator Diseases Across Europe Study) demonstrated that routine testing of patients presenting with specific indicator conditions, where HIV prevalence exceeds 0.1%, is both effective and cost-effective [9]. This approach has been endorsed by ECDC, WHO, and incorporated into European AIDS Clinical Society (EACS) guidelines [10].

Mononucleosis-like illness (MONO) and community-acquired pneumonia (CAP) represent two important HIV indicator conditions. Primary HIV infection frequently manifests as an acute retroviral syndrome clinically indistinguishable from infectious mononucleosis [11], while recurrent pneumonia is an AIDS-defining condition and HIV-infected individuals have a 5–29 fold higher incidence of pneumonia compared to the general population [12,13].

**Fig 1:**
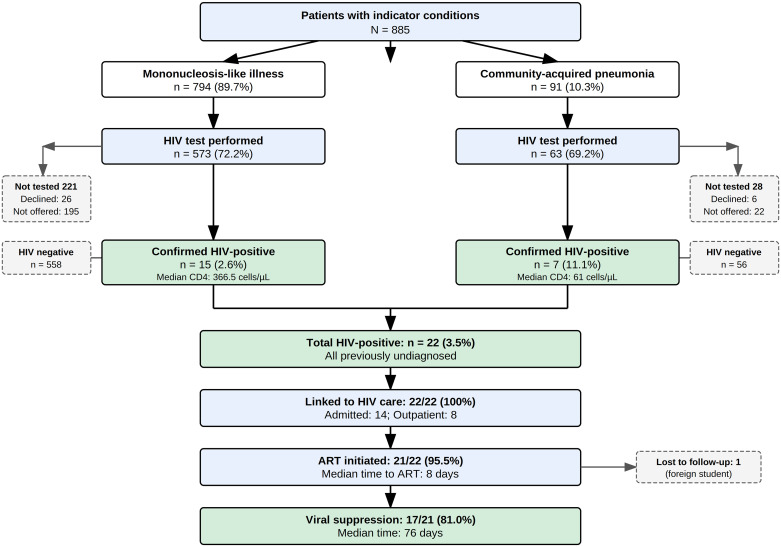
Study flow diagram. Flow diagram of patient enrolment, HIV testing uptake, and outcomes.

We previously reported that 58% of patients with mononucleosis-like illness referred to the Hospital for Infectious Diseases in Warsaw missed opportunities for HIV testing due to the absence of a systematic testing programme [14]. Despite endorsement at the European level, prospective data on IC-guided HIV testing remain scarce for Central and Eastern Europe, for Emergency Department settings specifically, and for the complete care cascade following diagnosis. The present study, conducted as part of the European OptTEST project, aimed to evaluate the implementation of IC-guided HIV testing among patients presenting with MONO and CAP in an Emergency Department setting, with the following outcomes: HIV test uptake, HIV seroprevalence, and linkage to HIV care including ART initiation and viral suppression (Fig 1).

## Materials and methods

### Study design and setting

This prospective cohort study was conducted at the Emergency Department of the Hospital for Infectious Diseases in Warsaw, Poland, from 10 May 2015 to 10 March 2018. The study was implemented as part of the OptTEST by EuroTEST project, aimed at optimizing HIV testing and linkage to care across Europe [15]. The Hospital for Infectious Diseases is the largest specialized infectious diseases hospital in Poland and serves as the principal referral centre for the Warsaw metropolitan area (population approximately 3.1 million), which is the rationale for selecting this single-centre setting.

### Participants

All patients aged 18–65 years presenting to the Emergency Department with a clinical diagnosis of mononucleosis-like illness or community-acquired pneumonia were eligible for inclusion. The age range of 18–65 years was defined by the OptTEST by EuroTEST study protocol and applied uniformly across all participating European centres. Exclusion criteria were a previously known HIV-positive status, inability to provide written informed consent, pregnancy, and recent hospitalization. Mononucleosis-like illness was defined as an acute febrile illness with pharyngitis, lymphadenopathy, and/or hepatosplenomegaly, based on clinical presentation and supported by a complete blood count with differential; serological testing for Epstein-Barr virus was performed in hospitalized patients, and the Paul-Bunnell heterophile antibody test was not used. Community-acquired pneumonia was diagnosed based on clinical presentation, physical examination, and radiological findings consistent with current guidelines [16]; chest radiography was required in all cases, and patients hospitalized within the preceding 14 days were excluded to rule out hospital-acquired pneumonia. Written informed consent was obtained from all participants.

### HIV testing and confirmatory procedures

All eligible patients were offered HIV DUO testing (Geenius HIV 1/2 Confirmatory Assay). Reactive results were confirmed using Western Blot (MP Diagnostics HIV Blot 2.2) and HIV RNA quantification (Abbott Real Time HIV-1). CD4 cell count was determined at diagnosis.

### Linkage to care

A standardized protocol was implemented to link patients with positive HIV DUO results to specialist care. Depending on clinical status, patients were either admitted to the hospital or scheduled for follow-up at the HIV Out-Patient Clinic. Data on time to ART initiation, viral suppression, and retention in care were collected.

### Data collection and management

Data were collected on demographics, indicator conditions, HIV test offer and uptake, test results, CD4 count, viral load, co-infections (HBV, HCV, syphilis), and linkage to care outcomes. Information on test offers and uptake was submitted electronically to the OptTEST coordinating centre via REDCap. Data collected at enrolment comprised demographics, clinical presentation, indicator condition, and HIV test result, with confirmatory testing, CD4 cell count, viral load, and co-infection screening obtained for HIV-positive patients. Follow-up data on linkage to care, ART initiation, and viral suppression were obtained retrospectively from HIV Out-Patient Clinic medical records, over a follow-up period of up to 12 months after diagnosis.

### Statistical analysis

Categorical variables were compared using the χ² test or Fisher’s exact test. Continuous variables were compared using Mann-Whitney U test because of the small and/or unequal subgroup sizes, the heterogeneous between-group variances, and the right-skewed distributions. HIV prevalence was calculated with 95% confidence intervals. Statistical significance was set at p < 0.05. All analyses were performed using IBM SPSS Statistics, version 25.0 (IBM Corp., Armonk, NY, USA). HIV prevalence was calculated among patients who accepted and underwent HIV testing (n = 636) rather than among all eligible patients.

### Ethical approval

The study was approved by the Bioethical Committee of the Medical University of Warsaw (approval number AKBE/35/15). Written informed consent was obtained from all participants prior to enrolment.

## Results

### Study population and test uptake

During the study period, 885 patients presented to the Emergency Department with the two indicator conditions: 794 (89.7%) with mononucleosis-like illness and 91 (10.3%) with community-acquired pneumonia. HIV testing was accepted by 636 patients (71.9% overall uptake), with similar acceptance rates between MONO (573/794, 72.2%) and CAP (63/91, 69.2%) groups. Among tested patients, 382 (60.1%) were male and 254 (39.9%) were female, with a median age of 26 years.

### HIV prevalence

Twenty-five patients had reactive HIV DUO tests. Of these, 22 (88%) were confirmed positive by Western Blot, yielding an overall HIV prevalence of 3.5% (95% CI: 2.2–5.2%). Prevalence was significantly higher among CAP patients (7/63, 11.1%; 95% CI: 4.6–21.6%) compared to MONO patients (15/573, 2.6%; 95% CI: 1.5–4.3%; p = 0.003). None of the HIV-positive patients had been previously diagnosed. Prevalence estimates use the number of patients tested as the denominator (n = 636) (Table 1).

**Table 1:** Characteristics of HIV-positive patients by indicator condition.

Characteristic	Total (n = 22)	MONO (n = 15)	CAP (n = 7)	p-value
Prevalence	3.5%	2.6%	11.1%	0.003
Age, years, median (range)*	32.5 (19-54)	31 (19-54)	38 (24-48)	NS
Male sex, n (%)	20 (90.9)	14 (93.3)	6 (85.7)	NS
MSM/bisexual, n (%)	13 (59.0)	11 (73.3)	2 (28.6)	0.026
CD4 count, cells/µL, median (range)	341 (4–731)	366.5 (88–731)	61 (4–450)	0.003
HIV VL, copies/mL, median (range)	272,210 (6,632–26,600,930)	587,904 (6,632–26,600,930)	272,210 (60,632–2,132,021)	NS
Hospital admission, n (%)	14 (63.6)	7 (46.7)	7 (100)	0.02
Time to ART, days, median (range)	8 (2–193)	15 (2–193)	6 (2–25)	NS
Viral suppression, n (%)	17/21 (81)	12 (85.7)	5 (71.4)	NS
**Continuum of care**				
Diagnosed with HIV, n	22	15	7	–
Linked to care, n (%)	22 (100)	15 (100)	7 (100)	–
Initiated ART, n (%)	21 (95.5)	14 (93.3)	7 (100)	NS
Achieved viral suppression, n (%)	17 (81.0)	12 (85.7)	5 (71.4)	NS
Time to viral suppression, days, median (range)	76 (14–356)	65.5 (14–356)	76 (46–133)	NS
Lost to follow-up, n (%)	1 (4.5)	1 (6.7)	0 (0)	NS

MONO, mononucleosis-like illness; CAP, community-acquired pneumonia; MSM, men who have sex with men; VL, viral load; ART, antiretroviral therapy; NS, not significant. MSM/bisexual includes homosexual (n = 12) and bisexual (n = 1) men. Viral suppression defined as HIV RNA < 50 copies/mL.

### Characteristics of HIV-positive patients

Among the 22 confirmed HIV-positive patients, 20 (90.9%) were male. Thirteen patients (59%) identified as MSM or bisexual (homosexual: 12, 54.5%; bisexual: 1, 4.5%), with a significantly higher proportion of homosexual men among MONO patients (73.3%) compared to CAP patients (14.3%; p = 0.026). The median age at diagnosis was 32.5 years (range: 19–54), with CAP patients being older (median: 38) than MONO patients (median: 31). HIV-positive patients were significantly older than HIV-negative patients (32.5 vs 26 years; p = 0.004), and male sex was significantly associated with HIV positivity (p = 0.005).

### Immunological status at diagnosis

The median CD4 count at diagnosis was 341 cells/µL (range: 4–731). There was a striking difference between indicator conditions: MONO patients had a median CD4 count of 366.5 cells/µL (range: 88–731), while CAP patients presented with severely compromised immunity (median 61 cells/µL; range: 4–450, p = 0.003). All seven CAP patients met criteria for late presentation (<350 cells/µL), compared to 8 of 15 (53%) MONO patients. Median HIV viral load was higher in MONO patients (587,904 copies/mL; range: 6,632–26,600,930) compared to CAP patients (272,210 copies/mL; range: 60,632–2,132,021), consistent with recent infection in the MONO group.

### Co-infections

Among HIV-positive patients, 2 (9.1%) had active HCV co-infection (anti-HCV and HCV RNA positive), 4 (18.2%) had evidence of past HBV infection (anti-HBc positive), 1 (4.5%) had chronic HBV (HBsAg positive), and 6 (27.3%) had positive VDRL tests indicating syphilis co-infection, all among MONO patients.

### Linkage to care and treatment outcomes

All 22 confirmed HIV-positive patients were successfully linked to care at the HIV Out-Patient Clinic. Fourteen patients (63.6%) required hospital admission due to severe illness, including all 7 CAP patients. Among CAP patients, Pneumocystis jirovecii was identified as the causative agent in 6 cases. Twenty-one patients (95.5%) initiated ART, with a median time from diagnosis to treatment initiation of 8 days (range: 2–193). One patient, a foreign student, was lost to follow-up. Among patients on ART, 17 of 21 (81%) achieved viral suppression, with a median time to suppression of 76 days (range: 14–356).

### Reasons for non-testing

Among the 249 patients (28.1%) who were not tested, only 32 (12.9%) actively declined testing, most commonly because they had been tested within the previous two years (during pregnancy or as blood donors). In 217 cases (87.1%), no documentation was available regarding why testing was not offered, predominantly occurring during night shifts.

## Discussion

This prospective study demonstrates that IC-guided HIV testing in an Emergency Department setting can identify a substantial number of previously undiagnosed HIV infections. The overall HIV prevalence of 3.5% among patients presenting with mononucleosis-like illness and community-acquired pneumonia was 35 times higher than the estimated national HIV prevalence in Poland (0.1%), strongly supporting routine HIV testing in this population [17].

The particularly high HIV prevalence among CAP patients (11.1%) is clinically significant and highlights CAP as a critical indicator condition. All CAP patients who tested HIV-positive presented with advanced disease and severe immunosuppression (median CD4 61 cells/µL), with Pneumocystis jirovecii identified in most cases. These findings are consistent with previous reports demonstrating that respiratory infections in HIV-infected individuals are often the first presentation of AIDS [12,13]. The 100% hospitalization rate among HIV-positive CAP patients underscores the clinical severity of these presentations and the importance of early HIV diagnosis before progression to AIDS-defining illnesses. This prevalence is consistent with, though higher than, the pneumonia-associated prevalence reported in the European HIDES I study [9], and likely reflects both the referral character of our centre and the predominance of Pneumocystis jirovecii pneumonia as an AIDS-defining presentation; the wide confidence interval (95% CI: 4.6–21.6%) warrants cautious interpretation given the small CAP subgroup.

In contrast, patients diagnosed through MONO presented with higher CD4 counts and very high viral loads, suggesting acute or recent HIV infection. This has important public health implications, as individuals with primary HIV infection and high viral loads are disproportionately responsible for HIV transmission [18]. Identifying these patients at the time of their acute retroviral syndrome provides an opportunity for both individual benefit and transmission prevention.

Our findings are particularly relevant in the context of recent epidemiological data from Europe. The 2024 ECDC/WHO surveillance report revealed that 48% of HIV diagnoses in the EU/EEA and 54% across the WHO European Region continue to be made at a late stage [1]. In Poland, recent studies have reported late presentation rates of 53–63% [6,7]. The persistence of high late diagnosis rates despite available effective treatment underscores the urgent need for expanded testing strategies.

The test uptake rate of 71.9% demonstrates good acceptability of routine HIV testing when offered in the context of indicator conditions. This is consistent with findings from other European studies showing that patients generally accept HIV testing when offered by healthcare providers [19,20]. Importantly, the main barrier to testing in our study was not patient refusal but failure to offer the test, particularly during night shifts. This finding highlights the need for systematic implementation of testing protocols that ensure all eligible patients are offered testing regardless of time of presentation.

The excellent linkage to care (100%) and rapid ART initiation (median 8 days) demonstrate that a well-structured testing programme can achieve optimal outcomes across the HIV care cascade. These results align with current recommendations for immediate or rapid ART initiation and suggest that ED-based diagnosis does not impede timely treatment initiation [21].

Several European countries have successfully implemented IC-guided testing strategies. In the United Kingdom, primary care physicians play an important role in HIV detection, and specialty guidelines increasingly recommend HIV testing for patients presenting with indicator conditions [22,23]. Similar programmes have been implemented in Spain, Denmark, and the Netherlands with positive results [24,25]. However, in Poland and many other Central and Eastern European countries, IC-guided testing is not routinely covered by public healthcare, resulting in continued missed opportunities for diagnosis.

This study has several limitations. First, it was conducted at a single specialized infectious diseases hospital, which may receive a higher proportion of patients with severe or atypical presentations compared to general hospitals. However, this also reflects a realistic setting where patients with suspected infectious aetiologies are commonly referred. Second, the study period predates the COVID-19 pandemic, which has had significant impacts on HIV testing and diagnosis across Europe [4]. Third, the relatively small number of CAP patients limits the precision of prevalence estimates in this subgroup. Fourth, eligibility was restricted to patients aged 18–65 years in accordance with the study protocol, which may have led to missed diagnoses among older adults, particularly in the CAP group, in whom pneumonia incidence rises with age.

Despite these limitations, our findings have important policy implications. The substantial yield of new HIV diagnoses (3.5% overall, 11.1% in CAP) far exceeds the 0.1% threshold recommended by European guidelines for routine HIV testing, providing strong justification for implementing IC-guided testing programmes in Polish healthcare settings [8]. The 2022 increase in HIV diagnoses in Poland, partly attributed to migration, further emphasizes the need for accessible testing strategies that can reach diverse populations [4].

From an implementation perspective, IC-guided testing entails modest resource requirements. The cost of a single HIV test is low relative to the lifetime cost of managing late-diagnosed HIV and its complications, and the HIDES studies demonstrated cost-effectiveness wherever HIV prevalence exceeds 0.1%, a threshold far exceeded in our population. In our experience, the protocol added only a few minutes per patient and could be integrated into existing Emergency Department workflows; the principal challenge was organizational, namely ensuring that testing was consistently offered across all shifts, including at night.

## Conclusions

This study demonstrates that IC-guided HIV testing among patients presenting with mononucleosis-like illness and community-acquired pneumonia in an Emergency Department setting is feasible, acceptable, and highly effective in identifying previously undiagnosed HIV infections. The HIV prevalence of 3.5% − 35 times higher than the national average – and near-complete linkage to care support the routine implementation of this testing strategy. Given the persistent challenge of late HIV diagnosis in Europe, including Poland, widespread adoption of IC-guided testing could significantly contribute to achieving the UNAIDS 95-95-95 targets and reducing HIV-related morbidity and mortality.

## Supporting information

S1 Checklist STROBE checklist for cohort studies. Completed Strengthening the Reporting of Observational Studies in Epidemiology (STROBE) checklist.
(DOCX)
